# The Effect of the Duration of Basic Life Support Training on the Learners' Cardiopulmonary and Automated External Defibrillator Skills

**DOI:** 10.1155/2016/2420568

**Published:** 2016-07-27

**Authors:** Jin Hyuck Lee, Youngsuk Cho, Ku Hyun Kang, Gyu Chong Cho, Keun Jeong Song, Chang Hee Lee

**Affiliations:** ^1^Department of Emergency Medicine, Hallym University School of Medicine, Seoul 24252, Republic of Korea; ^2^Department of Emergency Medicine, Samsung Medical Center, Sungkyunkwan University School of Medicine, Seoul 06351, Republic of Korea; ^3^Department of Emergency Medical Service, Namseoul University, Cheonan, Chungnam 331-707, Republic of Korea

## Abstract

*Background*. Basic life support (BLS) training with hands-on practice can improve performance during simulated cardiac arrest, although the optimal duration for BLS training is unknown. This study aimed to assess the effectiveness of various BLS training durations for acquiring cardiopulmonary resuscitation (CPR) and automated external defibrillator (AED) skills.* Methods*. We randomised 485 South Korean nonmedical college students into four levels of BLS training: level 1 (40 min), level 2 (80 min), level 3 (120 min), and level 4 (180 min). Before and after each level, the participants completed questionnaires regarding their willingness to perform CPR and use AEDs, and their psychomotor skills for CPR and AED use were assessed using a manikin with Skill-Reporter™ software.* Results*. There were no significant differences between levels 1 and 2, although levels 3 and 4 exhibited significant differences in the proportion of overall adequate chest compressions (*p* < 0.001) and average chest compression depth (*p* = 0.003). All levels exhibited a greater posttest willingness to perform CPR and use AEDs (all, *p* < 0.001).* Conclusions*. Brief BLS training provided a moderate level of skill for performing CPR and using AEDs. However, high-quality skills for CPR required longer and hands-on training, particularly hands-on training with AEDs.

## 1. Introduction

Bystander-initiated cardiopulmonary resuscitation (CPR) is critical to ensuring successful resuscitation in cases of out-of-hospital cardiac arrest [1]. Thus, layperson CPR training was initiated during the late 1970s by the American Heart Association, and many studies have highlighted that CPR education enhances the rates of bystander CPR [2, 3]. However, the bystander CPR rate remains relatively low and is a major obstacle to improving the survival rate for cases of out-of-hospital cardiac arrest [4, 5]. Several studies have indicated that a lack of CPR knowledge, anxiety regarding an adverse CPR outcome, and reluctance to perform mouth-to-mouth breathing all contribute to the low bystander CPR rate [6, 7]. Therefore, many efforts have been made to develop programs that provide basic life support (BLS) training. Recent studies have demonstrated the relative effectiveness of interactive computer- and video-based synchronous practice instruction, compared to conventional instructor-led courses [8, 9]. Furthermore, hands-only CPR is recommended for bystanders who are unwilling or unable to perform conventional CPR with mouth-to-mouth breathing [10]. Despite the effectiveness of BLS training for improving learners' performance during simulated cardiac arrest, there are few studies regarding the optimal duration of BLS training [11]. Thus, the present study aimed to compare the effectiveness of various durations of BLS training, based on the acquisition of CPR- and automated external defibrillator- (AED-) related skills, and the willingness and confidence of bystanders to perform CPR and apply an AED.

## 2. Materials and Methods

### 2.1. Study Design and Subjects

This study was funded by the Korean Centers for Disease Control & Prevention and approved by the institutional review board of Kangdong Sacred Heart Hospital. All participants were nonmedical college students who volunteered and provided their written informed consent. The participants received free BLS training between March 2015 and August 2015, as well as souvenirs that were worth approximately 10 American dollars.

During 2012, the Korean Centers for Disease Control & Prevention and the Korean Association of Cardiopulmonary Resuscitation (KACPR) developed four levels of BLS training for laypersons. For the present study, we randomly assigned participants to one of these four levels of BLS training: level 1 (hands-only CPR, 40 min), level 2 (hands-only CPR, 80 min), level 3 (conventional CPR, 120 min), and level 4 (conventional CPR, 180 min). Each course was administered to ≤35 participants, and a total of 16 courses (4 courses for each level) were provided during this study. Twenty-five instructors led these courses, and all instructors were doctors, nurses, or emergency medical technicians who were registered and certified as BLS instructors by the KACPR. All instructors were educated regarding the study's protocol. All BLS training courses were administered under instructor supervision, and training was provided using manikins and an AED-trainer; mouth-to-mouth breathing was performed using a face shield. All participants completed a questionnaire survey before and after each level of BLS training. At the end of each level, their CPR skills were also tested over 2 min using the Resusci Anne® manikin with Skill-Reporter software, and their AED-related skills were evaluated using a checklist. AED performance check list was authorized by KACPR. These tests were all supervised and conducted by certified BLS instructor.

### 2.2. BLS Course Format

The student-to-instructor ratio was 6 : 1, with at least one manikin and one AED for each group of two students. Each course was administered by first watching a video with the lead instructor and then practical training with a manikin (Little Anne®, Laerdal, Norway) and AED (AED Trainer2®, Laerdal, Norway). The specific contents of each program level are shown below and in Table 1:

- Level 1 (hands-only CPR, 40 min): introducing the participants to the course, showing a case of cardiac arrest, recognizing cardiac arrest and asking for help, teaching chest compressions, hands-on practice for hands-only CPR, introducing an AED and how to use it, introducing mouth-to-mouth breathing, and a course summary.
- Level 2 (hands-only CPR, 80 min): all level 1 contents, as well as hands-on practice with an AED and hands-on practice of hands-only CPR with an AED.
- Level 3 (conventional CPR, 120 min): all level 2 contents, as well as hands-on practice of mouth-to-mouth breathing, hands-on practice of conventional CPR, and hands-on practice of conventional CPR with an AED.
- Level 4 (conventional CPR, 180 min): all level 3 contents, as well as a BLS skill test using an AED, and providing feedback regarding CPR and AED skills.

### 2.3. Questionnaire

We modified a questionnaire from a previous study, which contained three parts: (a) the participant's demographics (age and sex) and experience with BLS training, (b) self-assessed confidence to perform bystander CPR and apply an AED, and (c) willingness to perform bystander CPR [6]. The self-assessed confidence to perform bystander CPR and apply an AED was evaluated using a visual analogue scale, with a score of 0 indicating “completely unable to perform CPR or use an AED” and a score of 100 indicating being “able to confidently perform CPR or use an AED.” The participants rated their willingness to perform CPR and use an AED using a 5-point Likert-type scale, with the scores categorized as “definitely no” (score 1), “no” (score 2), “not sure” (score 3), “probably yes” (score 4), and “definitely yes” (score 5).

### 2.4. Statistical Analysis

All statistical analyses were performed using SPSS software (version 19.0; SPSS Inc., Chicago, IL). Categorical variables were reported as number and percentage, and continuous variables were reported as mean and standard deviation (normal distribution) or median and interquartile range (IQR; nonnormal distribution). The willingness to perform CPR and use an AED before and after each level of the BLS training program was compared using the McNemar test, and confidence to perform CPR and use an AED was compared using the Wilcoxon signed rank test. Analysis of CPR quality among the four levels was performed using the chi-square test for categorical variables and using one-way analysis of variance (normal distribution) or the Kruskal-Wallis method (nonnormal distribution) for continuous variables. Post hoc paired comparisons between the levels were performed using the Mann-Whitney *U* test with Bonferroni corrections. All tests were two-tailed, and differences with a *p* value of <0.05 were considered statistically significant.

## 3. Results

### 3.1. Demographic Characteristics

A total of 502 participants signed up for and completed the BLS training. However, we excluded 10 participants who submitted incomplete questionnaires and 7 participants who had an error during the CPR quality check using the skill reporter. Therefore, 485 participants were included in the analyses. There was a significant, albeit small, difference when we compared the participants' ages for the various levels (21 years for levels 1-2 and 20 years for levels 3-4). However, there were no significant differences in the participants' sex, height, weight, body mass index, and prior BLS training. Among the 485 participants, 309 (63.7%) participants did not have prior BLS training experience (Table 2).

### 3.2. Analysis of CPR Quality according to Experience and Program Level

We analysed the CPR quality results among all participants and the novice participants. Among all participants, level 4 group had better CPR quality results (average compression depth and proportion of overall adequate compressions), compared to levels 1–3. There was no significant difference in the average compression rate, depth, and proportion of overall adequate compressions when we compared levels 1 and 2. However, the average compression depth exhibited a significant difference between level 3 (median: 51 mm, IQR: 44–57 mm) and level 4 (median: 55 mm, IQR: 50–59 mm; *p* = 0.003), and the proportion of overall adequate compressions also exhibited a significant difference between level 3 (median: 30.9%, IQR: 3.2–69.3%) and level 4 (median: 74.4%, IQR: 24.8–92.9%; *p* < 0.001). Post hoc paired comparison between levels 2 and 3 showed no difference in proportion of adequate compression depth (*p* = 0.451) and proportion of overall adequate compression (*p* = 0.119). The proportion of adequate recoils exhibited a significant difference among levels 1–4 (*p* = 0.006), and the post hoc paired comparison revealed a significant difference between level 1 and level 3 (*p* = 0.001). Only levels 3 and 4 involved mouth-to-mouth breathing, and there was no significant difference between level 3 (median: 25.0%, IQR: 0–44.4%) and level 4 (median: 20%, IQR: 0–50%; *p* = 0.825) (Table 3).

When we analysed only the novice participants, the proportion of overall adequate compressions exhibited a significant difference between level 3 (median: 29.8%, IQR: 2.4–67.8%) and level 4 (median: 56.7%, IQR: 9.8–88.9%; *p* = 0.008) (Supplemental Table  1 in Supplementary Material available online at http://dx.doi.org/10.1155/2016/2420568).

### 3.3. Analysis of AED Use according to Program Level

AED use was evaluated using a checklist at all steps for each level and between the various levels, with the highest performance observed in level 4. The correct location for the AED pads was observed for 85 (70.2%) participants in level 1, 107 (89.9%) participants in level 2, 114 (91.9%) participants in level 3, and 112 (92.6%) participants in level 4 (*p* < 0.001). The correct “immediate chest compression after shock” step was observed for 30 (24.8%) participants in level 1, 105 (88.2%) participants in level 2, 96 (77.4%) participants in level 3, and 110 (90.9%) participants in level 4 (*p* < 0.001). All participants successfully administered the shock within 90 s, although significant differences were observed when we compared to level 1 (median: 59 s, IQR: 55–65 s), level 2 (median: 53 s, IQR: 50–60 s), level 3 (median: 55 s, IQR: 50–60 s), and level 4 (median: 52 s, IQR: 48–56 s) (*p* < 0.001) (Table 4). The post hoc analysis revealed that level 1 participants exhibited poor performance for every step, except for “turn on the AED” step, compared to all other levels. The analysis of the novice group provided the same results as the analysis of all participants.

### 3.4. Effect of BLS Training on Willingness and Confidence to Perform Bystander CPR and Use an AED

The willingness to perform bystander CPR increased after BLS training, although there was no significant difference between the various levels. The willingness to use an AED also increased after BLS training, although there was no significant difference between the various levels. The self-assessed confidence in performing bystander CPR and using an AED increased after BLS training, although there was no significant difference between the various levels (Table 5). The analysis of the novice group provided the same results as the analysis of all participants.

## 4. Discussion

Our results confirmed that a relatively short duration of training improves CPR quality and the bystander's attitude towards CPR and AED use, although a longer duration of training was needed to achieve optimal CPR quality and AED use. Similarly, several previous studies have reported that relatively short durations of training improved CPR quality. For example, Hirose et al. reported improvements in CPR quality after a 45 min CPR training program using a personal manikin [12]. In the present study, level 1 (hands-only, 40 min) achieved an average compression rate of 122/min and a median compression depth of 51 mm, although the median of proportion of overall adequate compressions was 27.0%, which was lower than those for the other levels. This difference may be due to improper positioning of the chest compression site. In contrast, Panchal et al. evaluated ultrabrief video training and found that the median compression depth was 37 mm, which was not significantly different from that in their control group [13]. This difference may indicate that BLS training is limited when it is not led by an instructor and that there is some baseline training duration that is needed to achieve a significant improvement. For example, levels 1 and 2 provided similar CPR quality-related outcomes, although level 2 provided better AED use scores. Thus, level 2 may be more appropriate for general training regarding CPR and AED use.

When we compared levels 3 and 4 (120 min and 180 min of conventional CPR), we observed that level 4 provided better CPR quality-related outcomes. Similarly, Andresen et al. have reported that a longer duration of training improves CPR quality [14]. In this context, level 3 included 70 min of hands-on training, and level 4 included 100 min of hands-on training; the 30 min difference included hands-on skills testing after completing the training in level 4. This additional testing and feedback appear to have helped achieve a significantly higher proportion of overall adequate compressions in level 4 (74.4%), compared to those in the other levels. Interestingly levels 2 and 3 showed similar CPR quality outcome. We assume that level 3 group had to learn mouth-to-mouth breath in their given time which could distract their concentration but, level 2 group can concentrate their skill on only compression. Therefore, these results suggest that prolonged hands-on practice and immediate instructor feedback help improve the quality of chest compressions after completing a BLS course.

Many recent studies' results support the use of hands-only CPR for out-of-hospital CPR [15–17]. This is because hands-only CPR is as effective as conventional CPR and also because mouth-to-mouth breathing is a major barrier to bystander performance of CPR [6, 7]. Furthermore, our results indicate that levels 3 and 4 (conventional CPR training) achieved a poor quality of mouth-to-mouth breathing, even after 45 min of hands-on practice, and that the proportions of adequate mouth-to-mouth breathing were similar to the proportion in the novice group. This finding may indicate that it is important to emphasise hands-only CPR for laypersons, as a substantial amount of time would likely be needed to acquire an optimal level of skill in performing mouth-to-mouth breathing.

In the present study, we found that level 4 provided the highest score in applying an AED. In contrast, level 1 provided significantly lower scores in every step, with the exception of turning on the AED. This difference is likely related to the training in each level, as level 1 only introduced the participants to AEDs and did not include hands-on practice. Furthermore, many of the participants in level 1 did not say “clear” during their analysis and before the shock, which could lead to accidents when using AEDs, and three-quarters of the participants forgot to perform chest compressions after administering the shock. Although AEDs are intended for use by untrained laypersons [18], a short introduction without any hands-on practice does not appear to provide sufficient training for novices to adequately use these devices. Moreover, Gonzalez et al. have reported that two-thirds of laypersons can identify an AED and its purpose, although only approximately 50% of laypersons are willing to use AEDs [19]. Therefore, these results may indicate that relatively short BLS training with hands-on practice of AED is needed for laypersons to achieve satisfactory performance.

The present study demonstrated that improvements in willingness and confidence regarding CPR and applying an AED were independent of the BLS training duration. Similarly, Hirose et al. have reported that a simplified CPR training significantly increased the participants' confidence in performing CPR and applying an AED after the training [12]. Another study has demonstrated that even self-training (by watching a 22 min video) improved willingness and confidence regarding bystander CPR [20]. Therefore, relatively short BLS training may be sufficient to improve the willingness to perform bystander CPR among the general public.

## 5. Study Limitations

This study has several limitations that warrant consideration when interpreting our findings. First, every BLS course was administered by a qualified BLS instructor, but different combinations of the instructors were assigned to the same level at each time. Therefore, the emphasis on the training content may have varied according to each instructor's lecturing style, although we attempted to minimise this effect by training the instructors regarding each level's timeline. Second, the participants were all college student who were in their early twenties, which may have made them more capable of acquiring CPR and AED skills and more motivated to receive BLS training, than individuals from the general population. Therefore, the appropriate duration of BLS training may vary according to the laypersons' demographic characteristics (e.g., age and education level).

## 6. Conclusions

Our results indicate that a relatively short duration of BLS training helped the participants acquire CPR- and AED-related skills. However, a longer duration of training and hands-on practice was needed to achieve a high quality of skills for performing CPR and using AEDs. Furthermore, BLS training increased the participants' willingness and confidence to perform bystander CPR and to use an AED during cardiac arrest, regardless of the training duration.

## Supplementary Material

Comparing cardiopulmonary resuscitation among novice participants according to program level.

## Figures and Tables

**Table 1:** Comparing hands-on practice time for basic life support training courses according to program level.

Hands-on practice	Program level			
	1	2	3	4
Chest compression	10 min	15 min	10 min	10 min
AED	None^†^	15 min	15 min	15 min
Hands-only CPR with AED	None	15 min	None	None
Mouth-to-mouth breathing	None^†^	None^†^	10 min	10 min
Conventional CPR	None	None	25 min	25 min
Conventional CPR with AED	None	None	10 min	10 min
Skill test for CPR and AED	None	None	None	30 min

Total hands-on practice time	10 min	45 min	70 min	100 min
Total course time	40 min	80 min	120 min	180 min

The manikin-and-AED-to-student ratio was 2 : 1.

^†^Only introduction was provided by video and the lead instructor.

AED: automated external defibrillator; CPR: cardiopulmonary resuscitation.

**Table 2:** The characteristics of the 485 participants.

Characteristics	Program level				*p* value
	1 (*n* = 121)	2 (*n* = 119)	3 (*n* = 124)	4 (*n* = 121)	
Age (y/o)	21.0 (19.0–22.0)	21.0 (19.0–22.0)	20.0 (19.0–21.0)	20.0 (19.0–22.0)	0.003
Sex (male)^†^	55 (45.5%)	49 (41.2%)	52 (41.9%)	46 (38.0%)	0.707
Height (cm)	168.0 (162.5–175.0)	168.0 (162.0–174.0)	169.0 (163.0–174.8)	168.0 (163.0–175.0)	0.764
Weight (kg)	59.0 (53.0–70.0)	56.0 (51.0–68.0)	57.0 (52.0–66.8)	57.0 (51.0–68.0)	0.616
Body mass index (kg/m^2^)	20.8 (19.6–23.2)	20.4 (19.2–22.2)	20.2 (19.2–22.1)	20.5 (19.3–22.1)	0.205
Prior BLS training^†^ (yes)	48 (39.7%)	34 (28.6%)	46 (37.1%)	48 (39.7%)	0.231

All values were calculated using the Kruskal-Wallis method and expressed as median (interquartile range).

^†^Chi-square test (*n*, %).

BLS: basic life support.

**Table 3:** Comparing cardiopulmonary resuscitation quality among all participants according to program level.

Quality variables during CPR	Program level				*p* value		
	1 (*n* = 121)	2 (*n* = 119)	3 (*n* = 124)	4 (*n* = 121)	Between 1 and 2^†^	Between 3 and 4^†^	Among 1–4^‡^
Number of total compressions	225.0 (207.0–243.0)	216.0 (196.0–232.0)	150.0 (129.2–150.0)	148.0 (124.5–151.0)	0.018	0.734	<0.001
Average compression rate (per min)	122.0 (113.5–130.5)	120.0 (109.0–126.0)	120.0 (114.0–126.7)	119.0 (113.5–125.0)	0.037	0.467	0.110
Average compression depth (mm)	51.0 (46.0–56.0)	52.0 (46.0–56.0)	51.0 (44.0–57.0)	55.0 (50.0–59.0)	0.367	0.003	0.001^*∗*^
Proportion of adequate compression depth (%)	63.2 (19.0–97.9)	82.5 (25.5–98.6)	68.1 (8.0–98.7)	91.7 (45.2–98.7)	0.252	0.023	0.053
Proportion of adequate recoil (%)	100.0 (98.9–100.0)	100.0 (99.5–100.0)	100.0 (100.0–100.0)	100.0 (99.3–100.0)	0.050	0.170	0.006
Proportion of overall adequate compression (%)	27.0 (2.8–76.1)	42.7 (8.8–85.6)	30.9 (3.2–69.3)	74.4 (24.8–92.9)	0.094	<0.001	<0.001^*∗∗*^
Number of mouth-to-mouth breaths	—	—	8.0 (8.0–10.0)	8.0 (8.0–10.0)	—	0.368	—
Average ventilation volume (mL)		—	591.0 (326.0–886.0)	648.0 (354.5–902.5)	—	0.733	—
Proportion of adequate mouth-to-mouth breathing (%)	—	—	25.0 (0–44.4)	20.0 (0–50.0)	—	0.825	—
Hands-off time (s)	15.0 (13.0–17.0)	15.0 (13.0–17.0)	52.0 (48.0–56.0)	50.0 (47.0–55.5)	0.420	0.093	<0.001

^†^Post hoc paired comparisons between the levels were performed using the Mann-Whitney *U* test with Bonferroni corrections (statistical significance was *p* < 0.0083).

^‡^Calculated using the Kruskal-Wallis method.

^*∗*^Post hoc paired comparisons between levels 1 and 4 (*p* < 0.001) and levels 2 and 4 (*p* < 0.006).

^*∗∗*^Post hoc paired comparisons between levels 1 and 4 (*p* < 0.001).

CPR: cardiopulmonary resuscitation.

**Table 4:** Comparing automated external defibrillator application among all participants according to program level.

		Program level				*p* value
		1 (*n* = 121)	2 (*n* = 119)	3 (*n* = 124)	4 (*n* = 121)	
Turn on the AED	Correct	115 (95.0%)	114 (95.8%)	119 (96.0%)	120 (99.2%)	0.094
Correct location of AED pads	Correct	85 (70.2%)	107 (89.9%)	114 (91.9%)	112 (92.6%)	<0.001
Clear during analysis	Correct	50 (41.3%)	104 (87.4%)	104 (83.9%)	110 (90.9%)	<0.001
Clear before shock	Correct	44 (36.4%)	95 (79.8%)	95 (76.6%)	108 (89.3%)	<0.001
Immediate chest compression after shock	Correct	30 (24.8%)	105 (88.2%)	96 (77.4%)	110 (90.9%)	<0.001
Time from AED arrival until shock^†^	—	59.0 (55.0–65.0)	53.0 (50.0–60.0)	55.0 (50.0–60.0)	52.0 (48.0–56.0)	<0.001

Comparisons were performed using the chi-square test.

^†^Calculated using the Kruskal-Wallis method and reported as median (interquartile range).

AED: automated external defibrillator.

**(a):** 

		Program level				Total (*n* = 485)	*p* value	
		1 (*n* = 121)	2 (*n* = 119)	3 (*n* = 124)	4 (*n* = 121)			
Prewillingness score for CPR	Yes	77 (63.6%)	58 (48.7%)	73 (58.9%)	75 (62.0%)	283 (58.4%)	0.802^†^	<0.001^‡^
Postwillingness score for CPR	Yes	118 (97.5%)	115 (96.6%)	124 (100.0%)	117 (96.7%)	474 (97.7%)	0.879^†^	
Prewillingness score for AED	Yes	46 (38.0%)	42 (35.3%)	48 (38.7%)	56 (46.3%)	192 (39.6%)	0.158^†^	<0.001^‡^
Postwillingness score for AED	Yes	117 (96.7%)	113 (95.0%)	123 (99.2%)	119 (98.3%)	472 (97.3%)	0.162^†^	

A Likert scale was used to categorize responses as no (1, 2, and 3) or yes (4 and 5).

^†^The chi-square test was used to compare each program level.

^‡^The pre- and postwillingness for each program level were compared using the McNemar test (all, *p* < 0.001).

CPR: cardiopulmonary resuscitation; AED: automated external defibrillator.

**(b):** 

	Program level				*p* value	
	1 (*n* = 121)	2 (*n* = 119)	3 (*n* = 124)	4 (*n* = 121)		
Preconfidence for CPR	55.0 (30.0–70.0)	50.0 (30.0–80.0)	50.0 (40.0–70.0)	50.0 (30.0–80.0)	0.533^†^	<0.001^‡^
Postconfidence for CPR	90.0 (80.0–100.0)	90.0 (80.0–100.0)	90.0 (80.0–100.0)	90.0 (80.0–100.0)	0.177^†^	
Preconfidence for AED	50.0 (15.0–70.0)	50.0 (20.0–70.0)	50.0 (20.0–60.0)	50.0 (30.0–70.0)	0.579^†^	<0.001^‡^
Postconfidence for AED	90.0 (70.0–100. 0)	90.0 (80.0–100.0)	90.0 (80.0–100.0)	90.0 (80.0–100.0)	0.167^†^	

^†^Program levels were compared using the Kruskal-Wallis method and expressed as median (interquartile range).

^‡^Pre- and postconfidence were compared using the Wilcoxon signed rank test (all, *p* < 0.001).

CPR: cardiopulmonary resuscitation; AED: automated external defibrillator.

## Abbreviations

AED: Automated external defibrillator
BLS: Basic life support
CPR: Cardiopulmonary resuscitation
IQR: Interquartile range
KACPR: Korean Association of Cardiopulmonary Resuscitation.
