# Supplementary material for: The Effect of the Duration of Basic Life Support Training on the Learners' Cardiopulmonary and Automated External Defibrillator Skills
Source: Biomed Res Int. 2016 Jul 27;2016:2420568. doi: 10.1155/2016/2420568 (PMC4978818; doi:10.1155/2016/2420568)
Supplement: Supplementary file 1 — Comparing cardiopulmonary resuscitation among novice participants according to program level. [file 2420568.f1.pdf]

Supplemental Table 1: Comparing cardiopulmonary resuscitation among novice participants according to program level

Quality variables during CPR	Program level				P-value		
	1 (n = 73)	2 (n = 85)	3 (n = 78)	4 (n = 73)	Between 1 and 2†	Between 3 and 4†	Among 1–4‡
No. of total compressions	224.0 (206.5-237.0)	217.0 (195.5-231.0)	142.0 (127.7-151.0)	137.0 (121.0-150.0)	0.118	0.289	<0.001
Average compression rate (per min)	122.0 (113.0-130.5)	121.0 (109.5-126.0)	120.5 (113.0-126.3)	117.0 (111.5-123.5)	0.194	0.263	0.27
Average compression depth (mm)	49.0 (44.0-54.5)	51.0 (45.0-55.0)	51.0 (44.0-56.0)	55.0 (48.0-58.0)	0.436	0.029	0.019*
Proportion of adequate compression depth (%)	38.6 (16.3-90.4)	58.5 (17.2-94.7)	50.7 (7.3-93.9)	80.2 (22.6-98.2)	0.249	0.065	0.143
Proportion of adequate recoil (%)	100.0 (99.5-100.0)	100.0 (99.5-100.0)	100.0 (100.0-100.0)	100.0 (100.0-100.0)	0.439	0.255	0.148
Proportion of overall adequate compression (%)	25.4 (1.3-76.1)	32 (7.5-78.2)	29.8 (2.4-67.8)	56.7 (9.8-88.9)	0.351	0.008	0.049
No. of mouth-to-mouth breaths	-	-	8.0 (8.0-9.3)	8.0 (8.0-10.0)	-	0.242	-
Average ventilation volume (mL)	-	-	572.5 (288.5-846.8)	574.0 (313.0-858.5)	-	0.848	-
Proportion of adequate mouth-to-mouth breathing (%)	-	-	25.0 (0-50.0)	14.3 (0-40.0)	-	0.163	-
Hands off time (s)	15.0 (13.0-17.0)	15.0 (12.0-18.0)	51.0 (47.0-56.0)	50.0 (46.0-55.0)	0.436	0.081	<0.001

† Post hoc paired comparisons between the levels were performed using the Mann-Whitney U test with Bonferroni corrections (statistical significance was $p < 0.0083$).

‡ Calculated using the Kruskal-Wallis method.

* Post hoc paired comparisons between levels 1 and 4 ($p < 0.003$)

Appendix 1: AED performance check list

AED Performance Check list

UNIT NO.

◆ Instructor Profile

Name		Sex	M / F	Date	2015. . .

◆ 1-Rescuer Adult AED skill Check list

Skill Steps	Core skill performed - AED	Performed Correctly (√)
1	Turn on the AED first	
2	Correct location of AED pads	
3	Clear during analysis	
4	Clear before shock	
5	Immediate chest compression after shock (within 1 second)	
6	Time from AED arrival until shock	sec
Instructor's Decision	Pass / Fail	Signature:

Korean Association of Cardiopulmonary Resuscitation
